# 

**Published:** 1919-03

**Authors:** 


### CONTENTS FOR THE MARCH ISSUE

EDITORIALS.

Poem Taps.......................................... 293

Health from Public View-point......................... 294

Dr. Martin's Suggestion.............................. 295

The Medical Letter Bag............................... 296

Diseased Teeth and Diseases.......................... 297

Sugar Treatment of Tuberculosis...................... 297

Medical Colleges..................................... 297

Hospitals in South America........................... 298

Proper Food Keeps Off Disease........................ 298

ORIGINAL ARTICLES.

Technique of Suprapubic Prostatectomy. Dr. G. Kolische 299

Blood Pressure in Tuberculosis. Dr. W. O. Wilkes...... 304

Relapse vs. Infection. Dr. Robert W. Barton............ 308

A New Incision for Appendectomy. Dr. Leigh F. Watson 310

The Medical Letter Bag. Dr. H. E. Martin of Hot Springs, Ark.......................................... 311

Medical Miscellany.................................... 312

Book Reviews.......................................... 316

Army News............................................ 318

Thank You............................................. 320

News Items............................................ 321

Personals ............................................ 322

Old Jokes............................................. 323

